# DNA-Grafted 3D Superlattice Self-Assembly

**DOI:** 10.3390/ijms22147558

**Published:** 2021-07-15

**Authors:** Shuang Wang, Xiaolin Xie, Zhi Chen, Ningning Ma, Xue Zhang, Kai Li, Chao Teng, Yonggang Ke, Ye Tian

**Affiliations:** 1 Institute of Marine Biomedicine, Shenzhen Polytechnic, Shenzhen 518055, China; wangshuang20201009@163.com (S.W.); kaili1991@cug.edu.cn (K.L.); 2 State Key Laboratory of Analytical Chemistry for Life Science, Nanjing University, Nanjing 210023, China; xiexiaolin@smail.nju.edu.cn (X.X.); mg1934032@smail.nju.edu.cn (Z.C.); dz1834021@smail.nju.edu.cn (N.M.); dg20240144@smail.nju.edu.cn (X.Z.); 3 Wallace H. Coulter Department of Biomedical Engineering, Georgia Institute of Technology and Emory University, Atlanta, GA 30322, USA; 4 Shenzhen Research Institute of Nanjing University, Shenzhen 518000, China; 5 College of Engineering and Applied Sciences, Nanjing University, Nanjing 210023, China

**Keywords:** DNA nanotechnology, self-assembly, superlattice, DNA tile, DNA origami

## Abstract

The exploitation of new methods to control material structure has historically been dominating the material science. The bottom-up self-assembly strategy by taking atom/molecule/ensembles in nanoscale as building blocks and crystallization as a driving force bring hope for material fabrication. DNA-grafted nanoparticle has emerged as a “programmable atom equivalent” and was employed for the assembly of hierarchically ordered three-dimensional superlattice with novel properties and studying the unknown assembly mechanism due to its programmability and versatility in the binding capabilities. In this review, we highlight the assembly strategies and rules of DNA-grafted three-dimensional superlattice, dynamic assembly by different driving factors, and discuss their future applications.

## 1. Introduction

Self-assembly could arrange individual components into ordered structure, this phenomenon exists widely in nature, such as the double helix structure of DNA, protein aggregation and folding, cell, or some living life, and so on, they are considered to be the result of molecular self-assembly [1]. Inspired by the nature phenomenon and the unceasing understanding for self-assembly rules, researchers try to take atomic/molecular/ensembles in nanoscale as building blocks to fabricate devices, the automatic assembly among building blocks will obtain new structure/material with specific properties, which breakthrough the limitation of traditional top-down processing technology and meet the human needs in information, life, electronic, material fields. The assembly rules and methodologies are of great significance and dominate the material science field.

Colloid is often used as a model of atomic system to reveal the crystallization rules and the role of nanoparticle in assembly process [2]. Colloid nanoparticles as building blocks are linked together by weak interactions such as van der Waals, hydrogen bonding, or others to surface-bound ligands such as DNA [3], block copolymer [4], or small molecule [5]. Colloid particles protected by DNA with exceptional programmability and addressability exhibit tremendous potential in superlattice assembly. DNA-grafted nanoparticle as “programmable atom equivalent” (PAE) has been used in probing colloid assembly systems for many years.

In order to realize the concept of designing novel nanomaterials by bottom-up self-assembly, some challenges must be solved. For instance, clearing the relationships between interparticle interaction, assembly environment, and superlattice products. This review aims to highlight the critical factors for DNA-grafted 3D superlattice self-assembly, covering the species of building blocks (composition, size and shape), assembly methods (ssDNA, DNA tile, DNA origami nano-structure), dynamic manipulability for 3D superlattice (design, chemical stimulus, physical stimulus), and their potential application in fabricating functional materials as well as current challenges in this field. The design paradigms in this review call for a move toward the realm of intelligent nanofabrication.

## 2. Co-Crystallization of Different Building Blocks

### 2.1. Cocrystallization of Homogeneous Building Blocks

Assembly of DNA-grafted 3D superlattice is composed of two pivotal elements: nanoparticles (NPs) core and DNA shell. The progress of chemical synthesis technique enables the chemical composition of NP core unrestricted; it can be metals, oxides, polymers, even biological molecule, which also allow for the integration of multiple functions like luminescent, catalytic, magnetic properties, and so on.

Metal NPs are commonly employed because it is facile to modify dense DNA on the surface of NPs via thiol chemistry and their plasmon resonances could monitor the assembly process more easily. Majority of PAEs in superlattice assembly have spherical and isotropic NPs core. As early as in 1996, Mirkin’s group tried to assemble 3D superlattice using spherical gold nanoparticles (AuNPs) as core, and single-strand DNA as shell. Although the random binding of DNA shell on the surface of AuNPs increases the difficulty in assembly of ordered 3D superlattice. Until 2008, Mirkin and Gang realized the assembly of ordered 3D superlattice by accurately controlling the programmed DNA hybridization [6,7]. After plenty of experiments and theoretical simulations, Mirkin’s group published the pioneering work in Science about programmable assembly of colloid nanoparticles in 2011 [8]. In this work, nine distinct colloidal crystals, forty-one superlattices with different crystalline parameters were successfully synthesized and six design rules were concluded, which present methodologies for subsequent tailoring of 3D superlattice.

**Figure 1:**
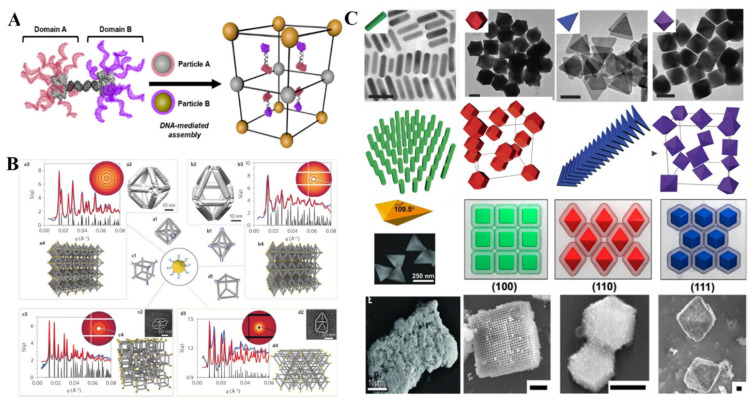
Strategies of control the valency and directional binding of PAE core by patchy or asymmetrically functionalized particles strategies (**A**) (Adapted with permission from [11]. Copyright 2018, American Chemical Society), anisotropic DNA origami framework (**B**) (Adapted with permission from [13]. Copyright 2012, Springer Nature.), and isotropic functionalization on the surface of non-spherical NPs (**C**) (Adapted with permission from [15]. Copyright 2016, American Chemical Society.) (Adapted with permission from [18]. Copyright 2017, Springer Nature.) NPs core with different shapes (top) and the corresponding lattice (bottom).

On the basis of spherical NPs core, researchers attempted to increase the complexity of PAE core in order to manipulate the superlattice assembly. Endowing the valency and specific directional bonding for NPs is effective in increasing the complexity of PAE core, and main three kinds of strategies of PAE construction were developed. First, patchy [9] or asymmetrically [10] functionalized particles strategies was proposed to control the number and direction of the DNA linkages. McMillan et al. used protein like beta-galactosidase to tune the DNA modification distributions, and then modulate the self-assembly of 3D superlattice [11] (Figure 1A). The numbers of amines and thiols on the protein surface determined the distribution of oligonucleotides, which directly guided the crystallization of nanoparticles to macroscopic superlattice. Further, they synthesized a protein-based Janus nanoparticle including two proteins tethered together by interprotein DNA hybridization [12]. The chemical anisotropy of such Janus non-centrosymmetric particle offers positions for asymmetric functionality and directional interaction. Employing this kind of novel particle, a unique hexagonal layered superlattice with unprecedented multicomponent nanoparticles was realized. This work is a paradigm of how to create highly engineered colloidal crystal by designing the building blocks. Second, anisotropic DNA origami structures dictate valency and directional binding to spherical NPs by trapping NPs within or at the vertices (Figure 1B). For instance, Tian et al. assembled the isotropic AuNPs into superlattice by taking DNA polyhedral frames as topological linkers [13]. Based on the different frameworks including octahedron, cube, and prism, different crystal forms like face-centered cubic, simple cubic, and simple hexagonal were fabricated successfully. They also challenged to build diamond-type lattice in virtue of the self-assembly of tetrahedron framework with low packing fraction, sensitivity to bond orientation, and heterogeneity [14]. The isotropic AuNPs associated at the vertices and center of tetravalent tetrahedron by DNA hybridization, forming face-centered cubic and diamond lattices. The isotropic functionalization on the surface of non-spherical NP is the third strategy to control valency and orientational binding of PAEs. In 2010, other anisotropic NPs like rods, triangular prisms, rhombic dodecahedra and octahedra directed the crystallization [15]. Later, NPs with eight different shapes (concave rhombic dodecahedra, cuboctahedra, concave rhombic dodecahedra, and so on) were obtained effectively via repetitive reductive growth and oxidative etching reaction [16] and were used to guide the lattice assembly. As a result, the low-symmetry and anisotropic NPs also exhibited the potential to form well-defined crystal habits, cube-shaped NPs formed a cubic habit, octahedron-shaped NPs formed a rhombic dodecahedron habit, and rhombic dodecahedron-shaped NPs formed an octahedron habit [17], even more complex clathrate crystals [18] without uniform habit formed from oblate trigonal bipyramids building block (Figure 1C).

To further enrich the lattice library, hollow spherical nucleic acid [19] NPs complexes as 3D spacer assembled to five distinct superlattices in symmetries including unprecedented crystalline material. Alkyne-modified DNAs were crosslinked on the surface of AuNPs and then the AuNPs template was dissolved to obtain the hollow particles which exhibit the same binding and assembly behavior as the DNA-modified AuNPs. Metal-organic frameworks (MOFs) are promising materials with well-defined porous structure, an abundance of chemical functionalities, widely applied in catalysis [20], gas storage and separation [21], and so on. In 2014, the first MOF NPs-DNA conjugates were created via click reaction between dibenzylcyclooctyne-functionalized DNA and azide-modified MOF material [22], and recently have been employed as building blocks of colloidal crystal engineering. For example, Wang et al. densely modified DNA on the surface of MOF NPs and assembled them into single-component MOF superlattice, binary MOF-Au single crystals, and 2D MOF nanorod superlattice [21].

Though assembly strategies and rules of NPs were deeply explored and developed, it is more challenging to create superlattice when the size of colloid increased to micrometer range. The relative thin DNA coating is difficult to rearrange further, which results in disordered aggregation. Crocker et al. introduced a block polymer spacer poly(ethylene glycol) to increase the length and flexibility of thin DNA to mediate the order crystallization of 1-μm diameter polystyrene particles [23]. Meulen et al. [24]. modified mobile DNA linkers on the surface of silica microparticles by integrating lipid bilayer, which could alleviate the challenges from sharp temperature window for equilibrium self-assembly and intrinsic surface heterogeneities. However, these two methods based on noncovalent modification are only fit for fabricating small or 2D crystals. Pine et al. employed strain-promoted alkyne-azide cycloaddition reaction to realize the high coverage of DNA on the surface of different micrometer-sized colloids with diverse chemical compositions such as organic poly(styrene), inorganic titania even a silica-methacrylate hybrid material, which could also be used to create ordered superlattice architectures. They explored different crystallization mechanisms and control the surface diffusion of micrometer-sized colloids and defects forming in the crystal [25,26]. As mentioned above, patchy functionalized protein was employed to synthesize a Janus NP [12], its valency and specific directional bonding could give some inspiration for assembly of homogenous NPs. These strategies in micro-meter colloid assembly gave inspiration of overcoming the problem in nanocrystal assemblies, promoting practical application in material fabrication.

### 2.2. Cocrystallization of Heterogeneous Building Blocks

**Figure 2:**
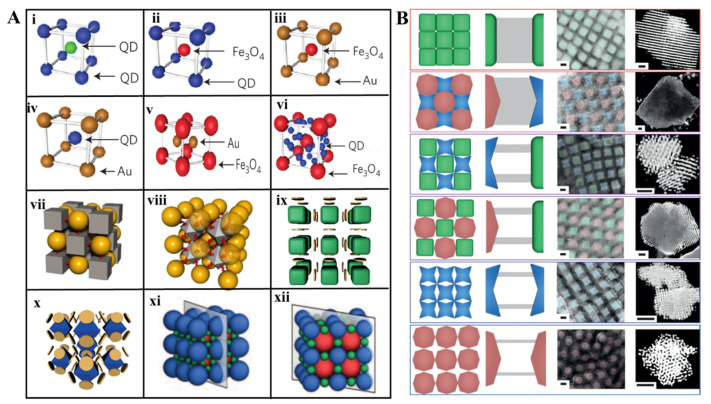
Assemblies of heterogeneous building blocks. (**A**) Crystallization of a binary system with same shape including two QDs with different sizes (**i**), arbitrary binary combination of QD, AuNPs, and Fe_3_O_4_ NPs (**ii**–**vi**) (Adapted with permission from [29]. Copyright 2013, Springer Nature); co-crystallization for hetero-shaped NPs including cube and sphere (**vii**), octahedron and sphere (**viii**) (Adapted with permission from [31]. Copyright 2015, Springer Nature.), cube and disk (**ix**), octahedron and disk (**x**) (Adapted with permission from [32]. Copyright 2015, Springer Nature.); crystallization of a ternary system composed of three AuNPs with different sizes (**xi**,**xii**). (Adapted with permission from [25]. Copyright 2013, American Association for the Advancement of Science.) (**B**) Size and shape complementary strategy for NP crystallization (Adapted with permission from [32]. Copyright 2015, Springer Nature.). From left to right: The packing model of shape complementarity; DNA connections between particles; high-magnification SEM and low-magnification STEM images of silica-encapsulated superlattices Scale bars, 50 nm.

Besides the wide range of superlattice structures assembled by the co-crystallization of homogeneous building blocks, researchers also tried to explore the assembly of different building blocks in order to integrate the functions of multiple materials. For instance, Park investigated the co-crystallization of a binary system including two same AuNPs with different sizes [6]. On the basis of the above pioneering work, other binary NPs systems were reported for the assembly of highly ordered lattices [27,28,29,30]. Sun et al. combined quantum dots (QDs) and gold nanoparticles(AuNPs) into a heterogeneous body-centered cubic lattice superlattice [27]. Zhang et al. synthesized lattices by arbitrary binary combination of QD, AuNPs, and Fe_3_O_4_ NPs [29] (Figure 2Ai–vi), in which the particle size, composition, interparticle distance, and crystal symmetry could be controlled independently.

Compared with assembly of spherical NPs by simple packing criteria, shape-induced directional bond for NPs crystallization is promising, but the co-crystallization for hetero-shaped NPs is much more difficult than spherical NPs mentioned above. Lu et al. achieved the cube-sphere and octahedron-sphere binary assembly (Figure 2Avii,viii) via the directional bonds [31]. By encoding the DNAs modified on the surface of different shape NPs, the hetero-shaped NPs are attractive and the same shape NPs are repulsive, which means the compatibility of DNA shell for shape disparity. Meanwhile, the spatial symmetry of block’s facets and NPs size ratio determine the lattice symmetry and structural order. O’Brien proposed size and shape complementary strategy for NP crystallization [32] (Figure 2B). They studied the effect of size complementarity on co-crystallization by two same cube NPs modified with DNAs of different lengths. Cube-shaped NPs with same size but with concave or convex faces binary system were used as a model to investigate the effects of shape complementarity on assembly. The assembly of cube-disk and octahedron-disk (Figure 2Aix,x) revealed that the number and type of directional interaction are closely related to the lattice arrangement.

Besides two building blocks were employed to engineer the 3D lattice mentioned above, increasing the diversity of lattice by integrating more than two building blocks is promising but challenging. So far, only a few works have overcome this obstacle. Mirkin’s group synthesized five ternary NPs superlattices by topotactic intercalation of the third NP component in binary lattice [28]. Furthermore, the interconversion between binary and ternary crystals were realized by modulating the temperature (Figure 2Axi,xii). When taking two NPs and two protein as building blocks, the work [12] of Hayes et al. in asymmetric functionality and directional interaction by chemical anisotropy of protein gave an example for lattice composed of more than two building blocks. In order to achieving the fabrication of metamaterial integrating multiple functions, more complex systems are highly desired.

## 3. Assembly Methods for 3D Lattice

### 3.1. Connection via ssDNA

**Figure 3:**
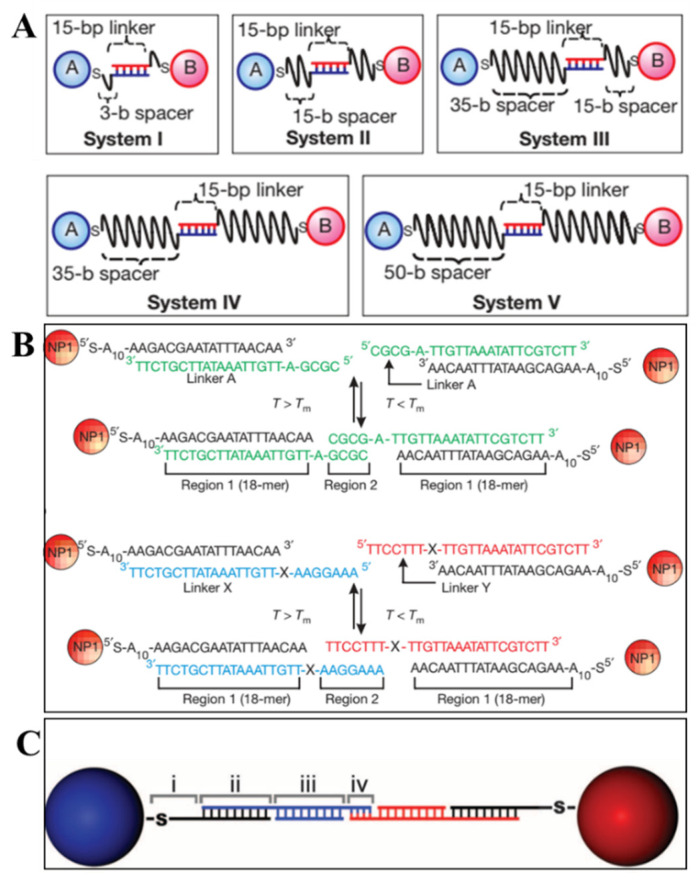
SsDNA linkages between NPs. (**A**) Connection by two linkers on the surface of NPs (System I to V: different spacers in length) (Adapted with permission from [7]. Copyright 2008, Springer Nature.); (**B**) connection by a free linker including a flexor region (X region) (Adapted with permission from [6]. Copyright 2008, Springer Nature.); (**C**) polyvalent conjugates of DNA and AuNPs by four free linkers. (Adapted with permission from [8]. Copyright 2011, American Association for the Advancement of Science.).

The simplest but typical method for 3D lattice assembly is two NPs connected to lattice by the complementary pairing of single strand DNA. In 2008, Gang’s group displayed the effect of different (linker and spacer) DNA structures on the long-range order of NP lattice [7]. The DNA linker in the middle offers driving force for A and B NPs assembly (Figure 3A). The length of linker sequence and the free spacer were designed to tune the attraction and repulsive interaction among NPs, which guide the transition from disordered NPs to 3D ordered lattice. According to the experimental results, the longer spacers (system IV and V) are beneficial for the long-range order of crystalline organization, and the shorter or more rigid spacers (system I–III) showed amorphous upon annealing. Besides, the melting temperature (Tm) of DNA linker sequence is a turning point for crystallization. The formation of lattice is kinetically prohibited when the temperature is much below Tm due to the local DNA crowding and high hybridization energy. While the DNA attraction energy will be reduced when the system is heated to Tm, which leads to the uniaxial DNA hybridization and distance uniformity between NPs and achieves optimal crystalline packing. The DNA design produces profound effect on forming ordered 3D lattice via the equilibrium balance of repulsion and attraction of assembly system. The difference of assembly method in Mirkin’s group [6] is the introduction of another linker DNA (Figure 3B) which includes two regions. The longer region is complementary with the DNA decorated on NPs, and the shorter region was designed to control the interaction among NPs and more thermally stable than the longer region. The design of these regions including length and sequence determined the effective radii of DNA-linked NPs, which also enable to modulate the competition relationship between entropic and enthalpic contributions in assembly process at different temperature. Additionally, a flexor region between above two regions is added to the linker. When deleting the single base adenine and adding a polyethylene glycol oligomer (PEG) to change the rigidity of flexor region, the latter formed a more ordered crystalline structure, which verifies that the greater flexibility is fit for crystallization. The controllable variations in connection method (as the more complex polyvalent conjugates of DNA and AuNPs, Figure 3C) aim at changing the hydrodynamic radii of DNA-NPs and the coordination environment of NPs, which are presented in one of the six rules for assembly of NPs superlattice [8]. Apart from the above factors, highly monodisperse NPs are also important.

### 3.2. Connection via DNA Tile

**Figure 4:**
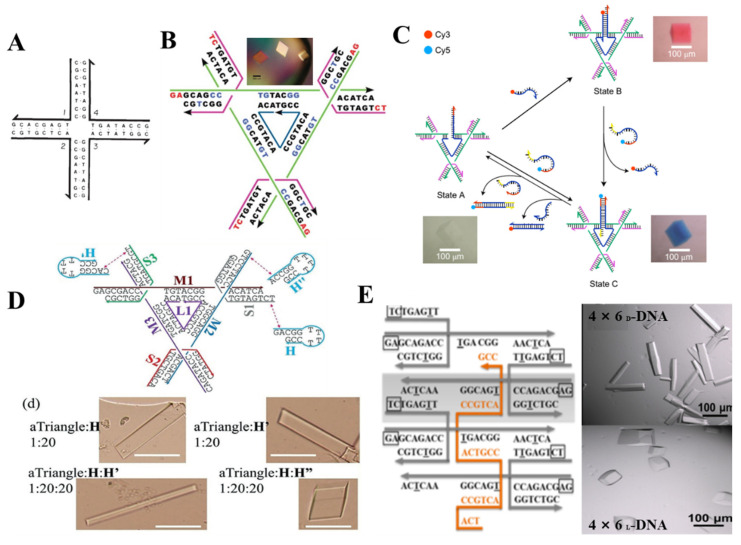
3D superlattice via DNA tile connection. (**A**) four-arm junction; (Adapted with permission from [33]. Copyright 1983, Springer Nature). (**B**) Design of tensegrity triangle as building block and the corresponding assembly, (Adapted with permission from [38]. Copyright 2009, Springer Nature.); (**C**) Three-state 3D device composed of 3D crystalline lattice and dynamic device (Adapted with permission from [39]. Copyright 2017, Springer Nature.); growth modulation for 3D lattice by agent strand in triangle motif (**D**) (Adapted with permission from [40]. Copyright 2018, WILEY−VCH Verlag GmbH & Co. KGaA, Weinheim, Germany). A weaving strand in 4 × 5 motif (**E**) (Adapted with permission from [41]. Copyright 2017, American Chemical Society).

DNA tile, derived from Holliday junction like the four-arm junction [33] in Figure 4A, consists of several stoichiometric single oligonucleotide strands. In order to assemble larger architecture, a crossover was introduced to design a rigid “DNA double-crossover (DX) molecule” as the building block. On both sides of the DX molecule, a single-strand DNA extended as sticky end for connecting other DX molecules. Based on the different DX motifs and above connection method, some 2D arrays [34,35,36], highly complex 3D nanostructures [37], were constructed and also employed to arrange NPs and proteins.

Though the position of NPs could be controlled precisely by DNA array or 3D DNA architecture assembled by DNA tile, there still are some obstacles to form a 3D crystal on the linear, planar template or even polyhedral frame. A groundbreaking work was published by Seeman that a single crystal was obtained by designing a tensegrity triangle [38]. This rigid triangle motif is composed of seven strands (three magenta strands form crossovers in the corners, three green strands extend for the length of each helix, one blue nicked strand at the center), in which the sequences of three magenta and green strands are same respectively, and the blue strand contains triply repeating sequence, a red short single-stranded sticky end was attached at the end of the six helices to connect with six other motifs, thereby yielding rhombohedral 3D crystal (Figure 4B). Afterward, they reported a three-state 3D device by combining devices and 3D crystalline lattice in one system [39] (Figure 4C). By designing the binding of coloring strand and central bule strand, the transition between blue and red crystal could be realized. Moreover, more efforts were made to modulate the growth, the cavity size and chirality, stability of 3D crystal by introducing an agent strand [40] (Figure 4D) at the end of the triangle motif, a weaving strand [41] in the center of a 4 × 5 motif (Figure 4E), enzymatic ligation [42] at position of the sticky end. To design 3D DNA crystals with higher resolution, a series of factors including sticky end length and sequences and 5′/3′ phosphates were systemically analyzed [43]. These advances make it feasible to extend 2D DNA array to 3D crystal, but the dimension of 3D crystal is still limited within a certain range, therefore more effective strategies are desired to break this obstacle.

### 3.3. Connection via DNA Origami Nanostructure

**Figure 5:**
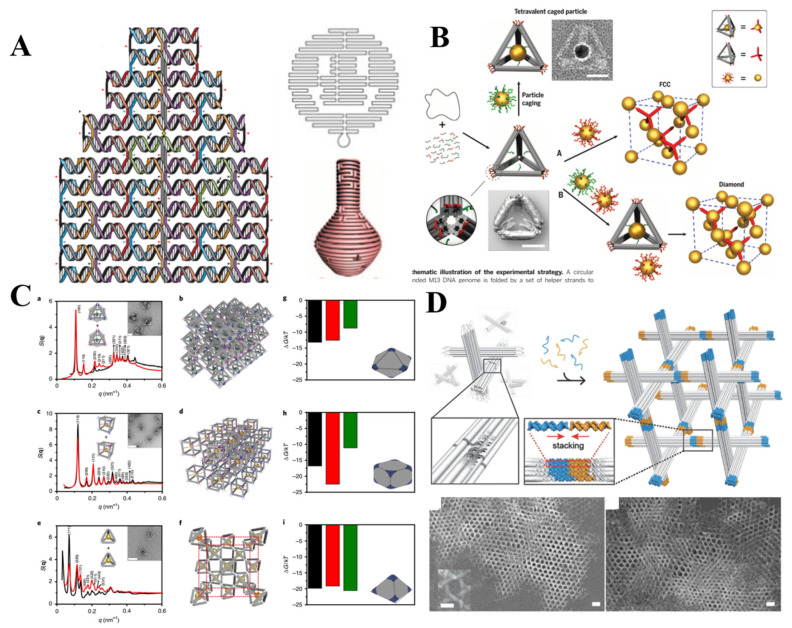
(**A**) Design of DNA origami and DNA origami-based simple 2D planar structure and intricate 3D architectures. (Adapted with permission from [44]. Copyright 2006, Springer Nature.) (Adapted with permission from [46]. Copyright 2011, American Association for the Advancement of Science); (**B**) face-centered-cubic and diamond lattice guided by DNA origami tetrahedron. (Adapted with permission from [14]. Copyright 2016, American Association for the Advancement of Science); (**C**) DNA origami tetrahedron, octahedron, cubic with different valances were employed to engineer diamond type, simple cubic and body-centered-cubic 3D AuNPs lattices. (Adapted with permission from [58]. Copyright 2020, Springer Nature.); (**D**) DNA origami-based structure assembled into 3D rhombohedral crystal. (Adapted with permission from [59]. Copyright 2018, WILEY-VCH Verlag GmbH & Co. KGaA, Weinheim).

DNA origami was first termed by Rothemund in 2006 [44], in which a long scaffold DNA with more than 7000 bases and hundreds of short staple strands assembled into anticipated structures ranging from simple 2D planar structure [44] to intricate 3D architectures [45,46,47] (Figure 5A). Over the past four decades, numerous design rules and assembly strategies have been developed to increase the complexity, advancing the structural DNA nanotechnology. The potential applications of these complex system turn to be of importance and attracts a lot of attention. For instance, DNA origami-based nanostructures were employed in biosensor [48,49,50,51], biocomputing [52,53,54,55], biomedicine [56] fields. Recently, DNA origami-guided 3D lattice engineering of inorganic NPs became one of the research hotspots.

Initially, NPs could be arranged to prescribed nanoclusters and low-dimensional arrays via the connection of the DNA frame origami structure [57]. A DNA origami octahedron frame is designed based on DNA origami technology, its six vertices can be encoded by extending the different sticky ends, thus determining the NP placement. The successful connection of DNA frame origami structure for 1D and 2D arrays meant it is possible to extend low-dimensional arrays to 3D lattice. One year later, Tian et al. realized above envision through designing the geometrical shapes and matching the topological connection among NPs by frame vertices. A series of DNA polyhedral frames (like cube, octahedron, elongated square bipyramid, prism, and triangular bipyramid) as topological linkers guided the assembly of 3D lattice with different lattice types and parameters such as simple cubic, face-centered-cubic, body-centered-tetragonal, simple hexagonal [13]. This approach sheds new light upon 3D lattice engineering. Liu et al. created a AuNPs diamond superlattice by tetrahedra with low packing fraction, orientation sensitivity and heterogeneity [14] (Figure 5B). The difficulty in diamond lattice assembly was successfully overcome by turning into the assembly between isotropic NP and anisotropic NPs with tetravalent binding topology.

Apart from connecting the NPs at the vertices of DNA frame, arbitrary positions within the frame could be encoded to anchor homogeneous or heterogeneous NPs. In 2020, Tian et al. connected NPs into the cavity of DNA frame structure to control the valence and coordination of NPs [58]. Compared with the typical ssDNA connection in NP lattice which is susceptible to the shell, size, and shape of NPs, this approach could be compatible with different objects even complex biomolecules. In the work, DNA origami frames with different valences (tetrahedra, octahedra, and cube with corresponding valence of 4, 6, 8), as material voxels were employed to engineer diamond type, simple cubic, and body-centered-cubic 3D AuNPs lattices (Figure 5C). Additionally, inorganic quantum dot and proteins including streptavidin, glucose oxidase, horseradish peroxidase were filled in the material voxels to expand the application of this assembly strategy.

Additionally, Liedl’s group assembled 3D crystal merely relying on DNA origami structure which have something in common with aforementioned tensegrity triangle designed by DNA tile [59]. This DNA origami-based tensegrity triangle structure is composed of three 14 helix bundles of 67 nm by interconnecting at defined positions (Figure 5D). The connection among triangular monomers relied on the shape complementary blunt-end stacking interaction. This three-fold symmetry DNA origami-based building block could assemble into a 3D rhombohedral crystal with 90% empty space which is larger enough to accommodate 20 nm NP, even ribosome-sized macromolecules. 

Above works give a good example for crystallization guiding by single DNA origami structure, but the co-crystallization for multiple anisotropic NPs is still challenging but attractive in engineering intricate materials. Tian’s group employed a couple of anisotropic DNA origami frames to alternatively encage NPs, creating a series of superlattice derivatives [60]. In this assembly system, crafting the positions and sequences for two kinds of building blocks (a regular and an elongated octahedra) determined the lattice parameter and type. Utilizing DNA origami building blocks to customize 3D lattice template allows hosting a wide variety of components and their properties, lays the foundation for metamaterial fabrication and structural biology application.

## 4. Dynamic Assemblies of 3D Lattice

DNA is regarded as an excellent candidate for engineering crystallization due to its high predictability, programmability by tuning the length, binding strength, and flexibility. Most obviously, the parameter of lattice could be modulated by the number of base pairs. Many factors in design influence the binding strength and flexibility and ultimately affect the crystallization ability and quality such as the sticky end sequences, number of the linkers, the complementary shape for blunt end, and so on. Some have been described in the previous section like the integrating ssDNA base or PEG “flexors” to tune the flexibility [6] and “agent” hairpin structure to control the orientation of crystal growth [40] (Table 1, top column).

The sensitive responsiveness of DNA for external chemical stimulus (Table 1, middle column) also promotes the development of dynamic lattice. Oh et al. demonstrated the dilatation-contraction in volume by the stabilization interaction of Ag^+^ with duplex in lattice [61]. Besides the reversible 25% dynamic change in volume, the stability is greatly improved that such Ag^+^-stabilized crystal did not dissociate when moving from water to organic media or air. Similarly, ruthenium coordination complexes [62], ethidium bromide [63] also increase the bond strength. Other multivalent cations [64] like Cu^2+^, Mn^2+^, Co^2+^, Ca^2+^, Na^+^, NH_4_^+^ actuated the NPs superlattice in varying degrees by screening the negative charges on the DNA backbones. Taking Ni^2+^ as a typical example, the volume change could reach as high as 65% by optimizing the concentration.

The osmotic pressure and dielectric constant of local solution environment could also modulate the superlattice structure dynamically. In 2013, Srivastava et al. explored the compression of 3D lattice in solution with different osmotic pressure modulated by polyethylene glycol of different concentrations [65]. The lattice constant remarkably decreased, corresponding to over 80% of the volume reduction. Later Mason et al. added ethanol to control the DNA bond length ranging from 3 to 16 nm, the expansion and contraction of crystals in volume reached by 75% while maintaining long-range order and the habit shapes [66]. Adding specific “i-motif ” sequence into the linker region endows the 3D lattice dynamic responsiveness, as the crystal exhibits conformational changes between condensed and extended states upon adjusting pH from low to high [67].

Mirkin’s group incorporated azobenzene-modified linking DNA to synthesize light-responsive NPs lattice [68]. The photoisomerization of azobenzene molecules as a driving force was employed to modulate the reversible assembly and disassembly of lattices with different crystal types. For PAE core with responsiveness for the external stimuli could also dictate the dynamic assembly property. For example, magnetic Fe_3_O_4_ NPs changed their orientation upon applying a magnetic field [69], accompanied by an unexpected crystallization alignment (Table 1, bottom column). The final superlattice is the consequence of competition between physical magnetic dipole–dipole and chemical DNA shell interaction. These dynamic manipulations for lattice display powerful potentials in manufacturing novel stimuli-responsive materials and devices, and more dynamic responsive systems have been highly desired.

## 5. Properties of DNA-Grafted 3D Lattice and Their Future Application

In recent decades, a variety of design rules and assembly strategies have been developed to engineer 3D superlattice with dozens of different symmetries. Most research has been done on two components (NP core and DNA shell) to broaden the crystal library. The successful design and assembly of 3D superlattice mentioned above will arguably bring innovative opportunities, the integration of structural DNA nanotechnology and dynamic assemblies of 3D lattice push applications further. Material fabrication with custom high-performance properties has been a long-term goal for NPs self-assembly and also there are enormous possibilities and challenges for such application. The lattice properties are inextricably linked with the lattice structure (for example, the atomic radius effect the electron affinity), while decoupling the interaction and distance between particles from composition and size of particles are important advantages for fabricating materials with novel plasmonic, photonic, electric, magnetic, mechanical, or catalytic properties.

DNA-mediated superlattice composed of noble-metal NPs exhibits potential to fabricate plasmonic metamaterials. Mirkin’s group first used electrodynamic simulation and experiment to prove that the superlattice is composed of spherical silver NPs (AgNPs) showing plasmonic properties [70]. Compared UV-visible spectroscopy of collective superlattice with individual NPs, a red-shifting is observed. In addition, binary AgNPs-AuNPs superlattice showed a distinctly dampened plasmonic response than the monometallic superlattice. The plasmonic function of superlattice is promising for the application in optical circuitry. Sun et al. found that the superlattice composed of octahedral NPs showed opposite polarization-dependent backscattering with the lattice composed of spherical NPs though having same body-centered cubic lattice symmetry and rhombic dodecahedron crystal habit [71]. This polarization-dependent property of 3D DNA lattice lays the foundation for polarization-associated optical device and electromagnetic field enhancement application. A 3D body-centered cubic AuNPs superlattice microcavity coupled with dye molecules was used as the testbed to study directional light emission [72]. The plasmon-exciton interaction strength between dye dipoles and surface plasmonic AuNPs could be modulated by controlling the position of dyes in subnanometer precision, which is in accordance with the results of electrodynamics calculations. This study gives a compelling example of applying 3D lattice into plasmonic devices.

Photonic crystal material could prohibit spontaneous emission of light [73], showing value in diverse applications like photoelectrochemical cells, optical transistor, and so on [74,75]. Rao et al. devised a hierarchical self-assembly strategy for the assembly of photonic crystal material [76]. A triblock patchy NP first associated to the tetrahedral cluster, then stacked into periodically structured crystal. But in this approach, it is difficult to ensure that the patchy NP selected staggered bond orientation with nearest NPs. He et al. demonstrated patch–patch adhesion with a steric interlock mechanism by using tetrahedral clusters with retracted sticky patches, ensuring appropriate staggered bond orientation [77]. Besides the most prevalent spherical colloids and block copolymers as building blocks, DNA origami-based 3D crystallization was proved promising for fabricating photonic crystal in theory and realized the fabrication of the complete photonic bandgap [78]. Though none of the photonic crystal material has been fabricated yet via DNA origami-based assembly strategy, the study offers theory support and guidance for obtaining photonic crystal in experiment.

The electric property of superlattice also attracts numerous attentions, but the DNA scaffold in DNA-grafted superlattice possesses low conductivity. How to integrate nanomaterial with high conductivity is an effective strategy. But the intrinsically susceptible DNA lattice ascribed to DNA is stable in a narrow range of conditions, which limits the organization of diverse nanomaterials. Mineralization for DNA superlattice with silica of excellent chemical and temperature stability could perfectly break through the above limit while maintaining the original 3D topology structure of lattice though exposing to extreme temperature, pressure, and harsh radiation condition [79]. Silica coating not only maintains the structure, but also supplies a robust template for other nanomaterial conversation. Shani et al. created 3D superconducting nanostructures by the bottom-up assembly method [80]. A cubic superlattice composed of octahedral DNA frames and NPs as template was first coated by silica to increase the stability of the soft lattice, then it was coated with superconducting niobium using e-beam evaporation. Such work provides a conceptually new strategy for constructing complex superconducting materials. On the basis of similar strategies, Gang’s group also created a periodic 3D silicon carbide (SiC) nanoscale architecture with dramatically enhanced electrical conductivity [79]. These studies are encouraging steps toward electronic application of 3D superlattice.

Most advances of magnetic NPs-based superlattice depend on hydrophobic surface ligands, typically the stability agents introduced in colloid synthesis. For instance, Zeng et al. exemplified that magnetic binary FePt and Fe_3_O_4_ NPs superlattice showed synergistically enhanced magnetic performance [81]. Only a few DNA-grafted 3D magnetic superlattice was created, but the magnetic properties is not explored [69]. With the help of DNA in tailoring the surface interactions, a more superior magnetic property will be obtained in the near future.

The mechanical property of superlattice is closely correlated with phonon/molecular/ion transport function, which possesses potential to be applied to chemoselective separation like ultrafiltration. Elasticity and hardness reflected the mechanical performance, which depended on the surface ligand of superlattice. Macfarlane’s group used DNA-grafted superlattice assembly to investigate the material mechanical behavior [82]. Due to the tunability of DNA via specific nuclease pairing, the modulus of superlattice could be tuned nearly two orders of magnitude. By altering the bond strength, the superlattice could be deformed when applying mechanical force. This study lays the foundation for developing deformable materials.

Catalytic properties of superlattice could promote the development of another application direction. For example, based on the catalytic property of enzyme, Tian et al. filled two kinds of enzymes glucose oxidase and horseradish peroxide into octahedron voxels, then co-assembled in a 3D lattice. The enzyme lattice displayed increased catalytic activity by the enzymatic cascade [58] in lattice mode than single enzyme system. Surface stabilizer-mediated compositionally heterogeneous nanocrystal superlattice with catalytic property have been reported [83,84]. For instance, binary Pt-Pd superlattice Au/Fe_3_O_4_ superlattice after postprocessing could catalyze the reduction of oxygen to water [82] and oxidation of carbon monoxide [84] at a faster rate than the homogeneous nanocrystal. Though the catalysis function of DNA-grafted superlatticehas not been employed to date, the catalytic application of DNA-grated superlattice will not be far.

To sum up, a significant amount of assembly experiences including NP cores, connection methods, and preliminary verification of 3D lattice functions in this review will offer new strategies for overcoming the difficulty in material synthesis. A full understanding of the 3D superlattice assembly by further investigating the assembly kinetics and arbitrarily controlling the crystal parameters like symmetry, the size, and shape of crystal habit are still required. Combining the fresh ideas of bottom-up DNA-grated 3D superlattice assembly with traditional postprocessing will be a promising direction of 3D lattice-based nanofabrication and further promote the application in real world.

## Figures and Tables

**Table 1:** Driving factors of dynamic assemblies of 3D lattice.

Strategies	Driving Factors	References
Design	Sticky end sequence	[6]
	Number and structure of linkers	[51]
	Complementary shape of blunt end	[59]
Chemical stimulus	Ruthenium coordination complexes	[62]
	Ethidium bromide	[63]
	Multivalent cations (Ag^+^, Ni^2+^, Co^2+^, Mn^2+^, Cu^2+^, Ca^2+^, Na^+^, K^+^, Mg^2+^, NH^4+^)	[64]
	Polyethylene glycol	[65]
	Ethanol	[66]
	pH	[67]
Physical stimulus	Light	[68]
	Magnetic field	[69]

## Data Availability

Not applicable.
